# Differences in Facial Emotion Recognition between First Episode Psychosis, Borderline Personality Disorder and Healthy Controls

**DOI:** 10.1371/journal.pone.0160056

**Published:** 2016-07-28

**Authors:** Ana Catalan, Maider Gonzalez de Artaza, Sonia Bustamante, Pablo Orgaz, Luis Osa, Virxinia Angosto, Cristina Valverde, Amaia Bilbao, Arantza Madrazo, Jim van Os, Miguel Angel Gonzalez-Torres

**Affiliations:** 1 Department of Neuroscience, University of the Basque Country, Leioa, Basque Country, Spain; 2 Department of Psychiatry, Basurto University Hospital, Bilbao, Spain; 3 Research Unit, Basurto University Hospital, Red de Investigación en Servicios de Salud en Enfermedades Crónicas (REDISSEC), Bilbao, Vizcaya, Spain; 4 Department of Psychiatry and Psychology, South Limburg Mental Health Research and Teaching Network, EURON, Maastricht University Medical Centre, Maastricht, The Netherlands; 5 King’s College London, King’s Health Partners, Department of Psychosis Studies, Institute of Psychiatry, London, United Kingdom; Central Institute of Mental Health, GERMANY

## Abstract

**Background:**

Facial emotion recognition (FER) is essential to guide social functioning and behaviour for interpersonal communication. FER may be altered in severe mental illness such as in psychosis and in borderline personality disorder patients. However, it is unclear if these FER alterations are specifically related to psychosis. Awareness of FER alterations may be useful in clinical settings to improve treatment strategies. The aim of our study was to examine FER in patients with severe mental disorder and their relation with psychotic symptomatology.

**Materials and Methods:**

Socio-demographic and clinical variables were collected. Alterations on emotion recognition were assessed in 3 groups: patients with first episode psychosis (FEP) (n = 64), borderline personality patients (BPD) (n = 37) and healthy controls (n = 137), using the Degraded Facial Affect Recognition Task. The Positive and Negative Syndrome Scale, Structured Interview for Schizotypy Revised and Community Assessment of Psychic Experiences scales were used to assess positive psychotic symptoms. WAIS III subtests were used to assess IQ.

**Results:**

Kruskal-Wallis analysis showed a significant difference between groups on the FER of neutral faces score between FEP, BPD patients and controls and between FEP patients and controls in angry face recognition. No significant differences were found between groups in the fear or happy conditions. There was a significant difference between groups in the attribution of negative emotion to happy faces. BPD and FEP groups had a much higher tendency to recognize happy faces as negatives. There was no association with the different symptom domains in either group.

**Conclusions:**

FEP and BPD patients have problems in recognizing neutral faces more frequently than controls. Moreover, patients tend to over-report negative emotions in recognition of happy faces. Although no relation between psychotic symptoms and FER alterations was found, these deficits could contribute to a patient’s misinterpretations in daily life.

## Introduction

Facial emotion recognition (FER) is the capacity to interpret the emotions of others based on facial expressions [1]. FER is considered crucial for non-verbal communication and for social functioning [2]. Emotion detection task involves the identification of an emotional stimulus without specifically labelling the affective state [3,4] and is considered a key component of non-verbal communication.

Individuals with schizophrenia have problems in the perception of emotional states throughout the disorder [5]. Emotion processing alterations in patients diagnosed with schizophrenia, assessed using FER tasks, are associated with poorer community functioning [6], decreased ability to live independently and inadequate function at work [7], and decreased interpersonal skills [8]. Schizophrenia may be related to a more specific disturbance in the procedure of a set of negative emotions including anger, disgust, sadness and/or fear [9]. If these alterations are present in patients in remission as well as in patients in the acute phase of illness [10], they may represent a trait rather than a state related to psychosis. In an attempt to clarify this question, attention has focused on social cognition skills in those suffering a first episode of psychosis (FEP), and in recent times in groups “at risk” of psychosis. Several studies have repeatedly reported social cognitive impairments in FEP [11] and “at risk” populations [12]. Furthermore, a number of studies did not find differences in the alterations between patients experiencing a FEP and those in a later stage of the disorder [6,13]. Alterations in FER may thus represent a possible endophenotype related to genetic risk and the development of psychosis [14].

Given the fact that psychotic symptoms occur transdiagnostically [15], it may be productive to study FER in relation to subclinical psychotic experiences in other diagnostic groups. For example, FER alterations have been associated with various clinical variables in bipolar disorder [16], alcoholism and substance abuse [17], depression [18] and posttraumatic stress disorder [19].

Likewise, borderline personality disorder (BPD) is associated with trait elevation of psychotic symptoms. About 20–50% of patients with BPD experience psychotic symptoms (hallucinations and paranoid ideation) comparable to those in patients with psychosis from a point of view of the phenomenology, emotional meaning and their maintenance [20]. BPD patients consequently may constitute an interesting group for comprehension of cognitive processes related to the development of delusions. In addition, there is substantial evidence to support the fact that BPD patients frequently interpret ambiguous or neutral facial expressions as negative [21,22].

The purpose of the present study was twofold. First, we wished to compare possible FER alterations in a sample of FEP and BPD patients in comparison with a group of healthy controls (HC) and to examine to what degree FER alterations in patients is valence-specific, or restricted to particular emotions. To determine this, we first compared a group of FEP and BPD patients with HC for their capacity to precisely recognize facial expressions of the essential emotions. Second, we investigated associations between FER and positive, negative, depressive and disorganized symptoms in these 3 groups.

## Materials and Methods

### Ethics Statement

The local ethics committee (Ethics Committee of Clinical Research of Basurto University Hospital) authorized the study design. We obtained written consent from all patients of the study.

### Sample

The recruitment procedures for this study were described previously [23]. Data were obtained from a convenience sample of patients with a FEP, admitted consecutively to the inpatient unit of Basurto University Hospital (HUB) from January 2010 to December 2014. BPD patients were enlisted at the Day Hospital of HUB and AMSA Clinic. HC were recruited from the general population in the same catchment area as the patients, through announcements. Controls did not report first-degree relatives with a psychotic disorder. Inclusion criteria were the following (for the three groups): age between 18 and 60 years, adequate capacity of speaking and understanding Spanish language; for FEP patients: treatment with antipsychotic medication < 1 year. The psychotic episode fulfilled DSM-IV-TR criteria for affective or non-affective psychotic disorder; for BPD patients: meeting DSM-IV-TR criteria for BPD in the absence of current psychotic disorder comorbidity (two of the patients had previous history of psychotic symptoms). Exclusion criteria for three groups were (a) current or past comorbid diagnosis of any neurological disorder which could prevent neuropsychological task performance, (b) history of severe head injury, (c) current severe medical conditions, (d) any current drug dependence and (e) unwillingness to participate.

Socio-demographic and clinical variables collected in the sample have been detailed in a previous paper [23].

### Instruments

Degraded Facial Affect Recognition Task (DFAR) [24] shows images of 4 different actors (2 male and 2 female) representing the 4 emotions: angry, happy, fearful, and neutral. The task consists of 64 items made up of 16 face presentations in each emotion category. The emotions are shown with 100% and 75% intensity to increment the difficulty of the task. Subjects have to press a button when they have recognized the emotion. Results are the proportion correctly recognized as neutral, happy, fearful, and angry emotions and the overall proportion correct. This test is programmed using E-Prime software [25].

The Benton Facial Recognition Test (BFRT) [26], is a measure of the ability to match non-emotional, unfamiliar faces. This task was used to rule out subjects scoring insufficiently on general facial recognition skill.

IQ: The short form of the Wechsler Adult Intelligence Scale (WAIS)–III [27] was administered for an indication of intellectual functioning (IQ), and included the following tests: ‘Block Design’, ‘Digit Symbol’, ‘Arithmetic’ and ‘Information’.

The Positive and Negative Syndrome Scale (PANSS) [28] was used to assess symptoms in patients. This scale contains one positive symptom subscale, one negative symptom subscale and one subscale of general psychopathology. Each item can be rated from 1 (absent) to 7 (extreme). A specific model has been formulated for the social cognitive on the basis of disorganized symptoms [29]. Research to date suggests a strong association between poor mental state attribution and disorganization symptoms [30]. Van der Gaag et al. [31] developed a more fine-grained model of symptoms. This model also captures disorganized symptoms. The positive, negative, and disorganized symptom factors of the Van der Gaag model were used in our analyses.

Structured Interview for Schizotypy—Revised (SIS-R) [32] was administered to assess subclinical psychotic symptoms in controls. It is a semi-structured interview that contains 20 schizotypal symptoms and 11 schizotypal signs. We reduced the item scores to the 3 dimensions of (1) positive (referential thinking, magical ideation, illusions, and suspiciousness), (2) negative (social isolation, social anxiety, introversion, and restricted affect), and (3) disorganization schizotypy (goal directness of thinking, loosening of associations, and oddness) [29].

The Community Assessment of Psychic Experiences (CAPE) [33] was used to assess positive, negative and depressive symptoms along a frequency scale (1 = never to 4 = nearly always) and a distress scale (1 = not distressed to 4 = very distressed) in the HC and BPD groups. The mean CAPE positive, negative and depressive score was the mean of the positive, negative and depressive symptom frequency scale, respectively.

### Analyses

In order to decrease differences between groups in IQ, we performed post-hoc analyses excluding from sample all subjects with IQ <70 (N = 8) and healthy controls with very high IQ (>130, N = 11). We have included analysis in whole sample as additional material (S1–S4 Tables).

The descriptive statistical analysis was based on frequency tables, and means and standard deviations (SDs). The Kolmogorov-Smirnov test was used to test for deviation from normality. ANOVA with Scheffe’s method for multiple comparisons was used to examine differences between the three groups in continuous variables, and Kruskal-Wallis test was used for non-normally distributed variables. For the comparison of categorical variables, chi-square tests, and Fisher´s exact test, when indicated, were performed.

In order to analyse differences between groups in emotion recognition and to compare the type of misinterpretation during FER among the three groups, the ANOVA with Scheffe’s test for multiple comparisons or the non-parametric Kruskal Wallis test was used. Further on, the general linear model was used to analyse the differences in emotion recognition and type of misinterpretation during FER between the three groups adjusting for WAIS, age and sex.

We carried out transversal within-group analyses. Associations between emotion recognition and symptom domain was analysed using the Spearman correlation coefficient.

Statistical analyses were carried out with STATA version 12 [34].

## Results

### Descriptives

64 FEP, 37 BPD and 137 controls were assessed at baseline. Subjects with Benton test scores of less than 41 were excluded due to insufficient general facial recognition ability.

Socio-demographic characteristics are shown in Table 1. CAPE data were missing for three BPD patients. Diagnoses in the FEP group were: schizophrenia or schizophreniform disorder (n = 32), affective psychosis (n = 18), brief psychotic episode (n = 7) and delusional disorder (n = 7). All FEP patients were under antipsychotic medication at the time of the assessment. BPD patients were under psychopharmacologic treatment as well. Clinical variables are summarized in Table 2.

**Table 1:** Socio-demographic variables.

*Variable*	*FEP patients (n = 64)*	*BPD patients (n = 37)*	*Controls (n = 137)*
Age, mean (SD)	35.5 (12.9)	36.8 (10.3)	33.1 (11.4)
Gender, n (%)*			
Male	41 (64.1%)	12 (33.3%)	77 (56.2%)
Female	23 (35.9%)	24 (66.7%)	60 (43.8%)
Education years, mean (SD)*	15.5 (3.2)	16.7 (2.7)	17.7 (2.5)
Partnership status, n (%)*			
Single	39 (60.9%)	21 (60%)	76 (55.5%)
Married/stable partnership	17 (26.6%)	10 (28.6%)	58 (42.3%)
Divorced/separated	6 (9.4%)	4 (11.4%)	3 (2.2%)
Widowed	2 (3.1%)	0 (0%)	0 (0%)
Housing, n (%)*			
With original family	35 (54.7%)	15 (42.9%)	58 (42.3%)
With own family	18 (28.1%)	12 (34.3%)	69 (50.4%)
Alone	11 (17.2%)	8 (22.9%)	10 (7.3%)
Employment status, n (%)**			
Full-time employment	31 (48.4%)	6 (17.1%)	78 (56.9%)
Unemployed	26 (40.6%)	25 (71.4%)	18 (13.1%)
Student	5 (7.8%)	1 (2.9%)	36 (26.3%)
Retired	2 (3.1%)	0 (0%)	2 (1.5%)
Other	0 (0%)	3 (8.6%)	3 (2.2%)
IQ, mean (SD)**	95.3 (14.4)	93.6 (12.5)	108.5 (12.7)

*p< 0.05

**p<0.0001

**Table 2:** Clinical variables.

	*FEP patients (n = 64)*	*BPD patients (n = 37)*	*Controls (n = 137)*
	mean (SD)	mean (SD)	mean (SD)
PANSS positive symptoms	20.3 (6)		
PANSS negative symptoms	12.2 (8.5)		
PANSS disorganized	10.9 (4)		
GAF score	56.5 (17.7)		
CAPE positive		11.5 (7.6)	4 (2.7)
CAPE negative		15.2 (7.8)	6.5 (4.1)
CAPE depressive		12.4 (5.9)	4.7 (2.4)
SIS-R positive			1.6 (1.7)
SIS-R negative			1.6 (1.3)
SIS-R disorganized			0.01 (0.1)

### Emotion Recognition

A significant difference between patient groups and HC on neutral face scores was found, as well as a difference on anger scores between HC and FEP patients. No significant differences were found between groups in the fear or happy conditions (Table 3). In general terms, patients with FEP and BPD displayed a lower ability in recognizing neutral faces. These results held true after adjusting for IQ, age and sex.

**Table 3:** Comparison of the percentage of correct answers for emotion recognition in FEP, BPD and HC. Unadjusted and adjusted analyses.

	Unadjusted analyses				
Emotion recognition	FEP^a^ (N = 64)	BPD^b^ (N = 37)	HC^c^ (N = 137)	Kruskal-Wallis test	
	mean (SD)	mean (SD)	mean (SD)	χ^2^_(df = 2)_	p-value
Neutral	78.3 (16.6)^c^	74.2 (14.4)^c^	83.9 (11.7)^a^^,^^b^	14.52	0.0007
Happiness	88.4 (11.5)	86.5 (13.5)	90.4 (9.7)	2.61	0.2709
Fear	50.7 (20.1)	56.1 (17.4)	53 (19.5)	1.96	0.3758
Anger	58.5 (22.2)^c^	65.2 (21.2)	68.1 (21.3)^a^	8.29	0.0158
Recognition	69 (11.5)^c^	70.5 (10.4)	73.8 (9.7)^a^	11.81	0.0027
	Adjusted analyses*				
Emotion recognition	FEP (N = 64)	BPD (N = 37)	HC (N = 137)	p-value	
	*β*	*β*	*β*	FEP vs. HC	BPD vs. HC
Neutral	-4.89	-8.48	Ref.	0.0343	0.0027
Happiness	-1.53	-3.48	Ref.	0.4012	0.1174
Fear	-0.26	3.48	Ref.	0.9347	0.3744
Anger	-8.84	-2.06	Ref.	0.0152	0.6406
Recognition	-3.88	-2.63	Ref.	0.0249	0.2092

^a,b,c^ Superscript letters indicate significant differences among groups by Scheffe test for multiple comparison.

* Comparison of the percentage of correct answers for emotion recognition between groups adjusting for WAIS, age and sex by means of the general linear models.

SD: standard deviation; df: degrees of freedom; β: beta parameter estimated from the general linear model, considering the healthy controls (HC) as reference group; Ref: Reference group.

### Errors in FER Answers

BPD y FEP groups had a much higher tendency to recognize negative emotions in happy faces. And BPD group had higher tendency to recognize also negative emotions in neutral faces as well. Moreover, after adjusting for other variables (IQ, sex and age), results remained significant (Table 4).

**Table 4:** Comparison of the percentage of subjects’ attribution when they failed in FEP, BPD and HC. Unadjusted and adjusted analyses.

	Unadjusted analyses				
	FEP^a^ (n = 64)	BPD^b^ (n = 37)	HC^c^ (n = 137)	Kruskal-Wallis test	
	mean (SD)	mean (SD)	mean (SD)	χ^2^_(df = 2)_	p-value
Neutral					
Happiness	10.7 (10.4)	12.4 (11.6)	10.9 (10.7)	0.57	0.7530
Negative valence	6.9 (8.5)	10.5 (10.5)^c^	5.7 (7.6)^b^	7.18	0.0276
Happiness					
Neutral	5.9 (7.1)	6.8 (7.5)	7.6 (7.8)	2.14	0.3439
Negative valence	3.3 (5.6)^c^	4 (7)^c^	1.5 (3.8)^a^^,^^b^	12.87	0.0016
Fear					
Neutral	33.3 (13.5)^c^	34.4 (15)	40.3 (15.1)^a^	11.42	0.0033
Happiness	3.6 (7.4)^c^	2.1 (3.3)	1.4 (3.3)^a^	10.95	0.0042
Anger	4.2 (6.6)	3 (5)	4.3 (6.2)	0.99	0.6097
Anger					
Neutral	18.4 (13.2)	12.9 (10)	16.9 (13.2)	3.84	0.1465
Happiness	2.8 (4.5)	3.3 (4.8)	3 (5.6)	1.07	0.5848
Fear	10.8 (10.3)	10.6 (10.9)	8.5 (10.4)	4.91	0.0858
	Adjusted analyses*				
	FEP (N = 64)	BPD (N = 37)	HC (N = 137)	p-value	
	*β*	*β*	*β*	FEP vs. HC	BPD vs. HC
Neutral					
Happiness	0.92	2.70	Ref.	0.6096	0.2200
Negative valence	0.53	3.47	Ref.	0.6856	0.0405
Happiness					
Neutral	-1.60	-0.74	Ref.	0.2122	0.6363
Negative valence	1.61	2.18	Ref.	0.0494	0.0288
Fear					
Neutral	-6.44	-4.41	Ref.	0.0097	0.1440
Happiness	2.10	0.73	Ref.	0.0094	0.4561
Anger	-0.92	-1.93	Ref.	0.3693	0.1249
Anger					
Neutral	0.96	-4.02	Ref.	0.6555	0.1274
Happiness	-0.64	0.07	Ref.	0.4642	0.9490
Fear	3.44	1.93	Ref.	0.0440	0.3513

^a,b,c^ Superscript letters indicate significant differences among groups by Scheffe test for multiple comparison.

* Comparison of the percentage of correct answers for emotion recognition between groups adjusting for WAIS, age and sex by means of the general linear models.

SD: standard deviation; df: degrees of freedom; β: beta parameter estimated from the general linear model, considering the healthy controls (HC) as reference group; Ref: Reference group.

FEP patients showed a significant tendency to recognize fear faces as neutral or happy.

### Association between Emotion Recognition and Symptom Domains

There was no significant association between emotion recognition and any of the different symptom domains in either patient group (correlation coefficients ranging from -0.23 to 0.22).

## Discussion

FER alterations have been reported for various mental disorders and may predict poor social functioning. What remains unclear is how specific these alterations are and if certain patterns characterize particular patient groups. This study examined disorder-specific emotional alterations in two groups of patients in a comparison with HC.

There were three main findings. First, our results showed poorer emotion recognition of neutral and angry faces in FEP and of neutral faces in BPD patients. Second, these alterations were not associated with (subthreshold) psychopathology. And finally, both patient groups tended to attribute emotional negative valence more frequently than HC to happy faces. Within the three groups, the level of positive symptoms was not associated with FER alterations. These FER alterations may be more prevalent in disorders with psychosis proneness rather than being associated to specific symptoms. This may imply that FER may be more a trait of psychosis rather than a state. However, it is possible that the statistical power of sample was not enough to detect such differences (maybe due to the low number of subjects, especially in the BPD group).

Nonetheless, this study demonstrates that patients who are prone to psychosis (BPD), process emotions similarly to patients with psychosis (FEP). These findings may serve to support the hypothesis that such biases are related to interpersonal disability of these patients observed clinically, although hardly any study found a relation between emotion recognition and social functioning in the daily life of psychotic patients [35]. We have described previously that FEP and BPD patients can share other cognitive biases, such as data gathering bias that may contribute to the formation of delusions [23].

### Facial Emotion Recognition Alterations

It is also still unclear whether deficits in facial emotion identification in people with psychosis are general or valence specific. Although one of the most consistent findings across studies are deficits on negative emotions (fear, disgust, sadness and anger) [14,36,37]; in our study, FEP patients only demonstrated poor recognition of angry faces, but not on fear emotion. In general terms, angry faces are more difficult to recognize [38] and this could be a possible explanation. However, this deficit is not enough to confirm our hypothesis about valence-specific deficit recognition. More relevant it seems the difficulty of recognizing neutral faces, such as other authors have described [39]. In the case of BPD patients, there is considerable experimental evidence to support the conclusion that ambiguous or neutral facial expressions are more likely to be interpreted negatively [21,22,40–43].

Our results indicate a worse FER in FEP and BPD patients, especially of neutral faces, and additionally of angry faces in psychotic patients. In general terms, we could state that there was poorer affect recognition of facial emotions on neutral emotion independently of cognitive performance in both groups of patients. These results suggest that patients with severe mental disorders display alterations in general emotion recognition.

Moreover, happy faces were interpreted as negative in both groups at much higher frequencies than in HC. This tendency was also significant for neutral faces in BPD group. Hence, the ability of deducing emotional states from others may be compromised in these patients [6]. Other studies documented that BPD patients present a tendency to over-report fear when they see a neutral facial expression [43]. Similarly, in the current study, BPD patients tend to interpret fear in happy and neutral faces more frequently than HC.

In general terms, until now the reported findings do not make it clear whether the impairment is selective for concrete emotions, is selected for valence, or affects the whole domain of basic emotions. In this study, there was a clear alteration in FER in BPD patients in the recognition of neutral emotion as well as a negative misinterpretation of happy faces in both patient groups.

Other authors [5,44,45] found a specific alteration for fear and sadness, but not for disgust and anger in psychotic patients. Additionally, Comparelli et al. [5] did not find any evidence of progression or improvement over the 3 phases of psychotic process (prodromal, FEP and multi episode psychosis). This finding is in agreement with the results of other authors [46], who recently showed, in a longitudinal study, absence of progression of FER alterations from the prodromal to the post-onset period, suggesting that FER represents a vulnerability trait for developing psychotic symptoms. We found a difficulty to recognize neutral and anger condition in FEP group. And as we have described, some of these FER alterations also appear in patients who are prone to psychosis (BPD patients).

Our results indicate an alteration in recognition specifically of neutral faces in both groups, which could lead to perceive neutral emotions as negative and this can influence social interactions. Alterations in emotion recognition, although perhaps to a lesser degree, may cause negative misinterpretations of social-emotional situations. And misperceptions may lead to the avoidance of social situations or contacts and lead to problematic social behaviour.

### Facial Emotion Recognition Alterations and Psychopathology

To study the nature of the associations between symptoms and social cognitive impairment in patients and HC, we performed a cross-trait within-group analysis. We did not find any association between symptom domains and FER alterations in FEP. The analyses also failed to reveal significant associations between symptom domains and psychopathology measures in BPD or HC.

A previous study suggested an association between disorganized and negative symptoms and social cognitive performance in patients diagnosed with a psychotic disorder and, to a lesser extent, subclinical negative symptoms in siblings of patients diagnosed with a psychotic disorder [47]. The lack of replication of previous findings indicates that more research is required before more definitive conclusions can be reached.

### Strengths and Limitations

To the best of our knowledge, this is the first study to compare FER alterations in FEP and BPD patients in a comparison with HC. Studying individuals with FEP helps to minimize the influence of variables such as institutionalization, the long-term effects of antipsychotics and social deterioration, and could avoid variability due to the course of the illness [48].

Our results are independent of IQ performance. This is consequent with the fact that performance on social intelligence tasks is not strongly influenced by IQ [49]; emotion recognition in psychosis may be a different cognitive aspect only modestly influenced by other cognitive skills.

BPD patients commonly suffer from other psychiatric comorbidities such as depression, PTSD or drug abuse. These disorders also relate with FER alterations. The possible effects of these comorbidities need to be taken into account as well [50]. Although patients with BPD frequently present other diagnoses that are clinically important, they are not crucial to the diagnosis of BPD itself. Thus, although a complete understanding of emotion perception in BPD would be better in a sample without these comorbid disorders, we have not included comorbidity of BPD in analyses due to the low number of patients in that group.

Another limitation of the study is that we did not examine possible effects of psychotropic medication on emotion recognition. Although there have been a few studies reporting mild effects of psychoactive medication on FER [51], the recent literature reports no significant impact of antipsychotic [52] or antidepressant medication [53] on emotion recognition.

Divergences among previous studies with respect to the accuracy of FER alterations may have been the consequence of several factors including the use of different stimulus materials (e.g. Ekman versus another set of stimuli, inclusion versus exclusion of neutral faces), differences in the intensity of the emotional expression, time limits and task instructions [54]. As Fertuck [55] et al. observe, previous studies were not uniform in the selection of potentially confounding factors such as educational level, source of patient recruitment (inpatient versus outpatient), comorbidity, psychotropic medication, and state levels of psychopathology, all of which may influence emotion and mental state evaluation.

In conclusion, FER is impaired in FEP and BPD patients, especially for neutral and angry faces. Moreover, patients tend to over-report negative emotions in happy faces. Alterations appear not only in FEP, but also in BPD, suggesting that discrepancies in the processing of integrated facial emotions may be related to transdiagnostic interpersonal hostility, and social functioning. This may suggest that differences in the patients’ higher order integration of emotional perceptions may be associated with the large variation in interpersonal dysfunction observed clinically.

## Supporting Information

S1 Table Socio-demographic variables. (PDF)

S2 Table Clinical variables. (PDF)

S3 Table Comparison of the percentage of correct answer of emotion recognition in FEP, BPD and HC. Unadjusted and adjusted analyses.
(PDF)

S4 Table Comparison of the percentage of subjects’ attribution when they failed in FEP, BPD and HC. (PDF)
