# Supplementary material for: Differences in Facial Emotion Recognition between First Episode Psychosis, Borderline Personality Disorder and Healthy Controls
Source: PLoS One. 2016 Jul 28;11(7):e0160056. doi: 10.1371/journal.pone.0160056 (PMC4965014; doi:10.1371/journal.pone.0160056)
Supplement: S1 Table — (PDF) [file pone.0160056.s001.pdf]

Table 1. Socio-demographic variables.

Variable	*Group*		
	FEP patients (n= 69)	*BPD patients (n= 40)*	*Controls (n= 148)*
Age, mean (SD)	35.4 (12.6)	35.9 (10.3)	33.1 (11.3)
Gender, n (%)*			
Male	44 (63.8%)	12 (30%)	87 (58.8%)
Female	25 (36.2%)	28 (70%)	61 (41.2%)
Education years, mean (SD)*	15.4 (3.2)	16.5 (2.7)	17.8 (2.4)
Partnership status, n (%)*			
Single	41 (59.5%)	23 (59%)	81 (54.7%)
Married/stable partnership	19 (27.5%)	11 (28.2%)	64 (43.2%)
Divorced/separated	7 (10.1%)	5 (12.8%)	3 (2.1%)
Widowed	2 (2.9%)	0	0
Housing, n (%)*			
With original family	38 (55%)	18 (46.2%)	63 (42.6%)
With own family	20 (29%)	13 (33.3%)	75 (50.7%)
Alone	11 (16%)	8 (20.5%)	10 (6.7%)
Employment status, n (%)**			
Full-time employment	33 (47.8%)	8 (20.5%)	84 (56.8%)
Unemployed	29 (42%)	27 (69.3%)	19 (12.8%)
Student	5 (7.3%)	1 (2.5%)	40 (27%)
Retired	2 (2.9%)	0	2 (1.4%)
Other	0	3 (7.7%)	3 (2%)
IQ, mean (SD)**	93.1 (15.9)	91.2 (14.6)	110.6 (14.4)

*p< 0.05
**p=0.0001
