# Supplementary material for: Differences in Facial Emotion Recognition between First Episode Psychosis, Borderline Personality Disorder and Healthy Controls
Source: PLoS One. 2016 Jul 28;11(7):e0160056. doi: 10.1371/journal.pone.0160056 (PMC4965014; doi:10.1371/journal.pone.0160056)
Supplement: S2 Table — (PDF) [file pone.0160056.s002.pdf]

Table 2. Clinical variables.

	FEP patients (n=69)	BPD patients (n=40)	Controls (n=148)
	mean (SD)	mean (SD)	mean (SD)
PANSS positive symptoms	20.5 (5.9)		
PANSS negative symptoms	12 (8.2)		
PANSS disorganized	10.7 (3.9)		
GAF score	56.6 (17.3)		
CAPE positive		11.8 (7.9)	4 (2.6)
CAPE negative		15.3 (7.5)	6.7 (4.2)
CAPE depressive		12.7 (5.8)	4.7 (2.4)
SIS-R positive			1.5 (1.6)
SIS-R negative			1.5 (1.3)
SIS-R disorganized			0.01 (0.1)
