# Supplementary material for: Differences in Facial Emotion Recognition between First Episode Psychosis, Borderline Personality Disorder and Healthy Controls
Source: PLoS One. 2016 Jul 28;11(7):e0160056. doi: 10.1371/journal.pone.0160056 (PMC4965014; doi:10.1371/journal.pone.0160056)
Supplement: S3 Table — Unadjusted and adjusted analyses. (PDF) [file pone.0160056.s003.pdf]

Table 3. Comparison of the percentage of correct answer of emotion recognition in FEP, BPD and HC. Unadjusted and adjusted analyses.

Emotion recognition	Unadjusted analyses				
	FEP[a] (N=69)	BPD[b] (N=40)	HC[c] (N=148)	Kruskal-Wallis test	
	mean (SD)	mean (SD)	mean (SD)	$\chi^2_{(df=2)}$	p-value
Neutral	78.9 (16.2)[c]	74.5 (14)[c]	84.1 (11.8)[a,b]	15.54	0.0004
Happiness	88.6 (11.2)	86.4 (13.3)	90.8 (9.6)	4.20	0.1225
Fear	50.8 (19.8)	54.4 (18.4)	53 (19.5)	1.26	0.5317
Anger	59.1 (21.9)[c]	65.2 (20.6)	68.2 (21.7)[a]	7.63	0.0220
Recognition	69.4 (11.4)[c]	70.1 (10.2)	74 (9.7)[a]	12.80	0.0017
Emotion recognition	Adjusted analyses*				
	FEP (N=69)	BPD (N=40)	HC (N=148)	p-value	
	β	*β*	*β*	FEP vs. HC	BPD vs. HC
Neutral	-4.91	-8.75	Ref.	0.0301	0.0014
Happiness	-1.26	-3.53	Ref.	0.4757	0.0991
Fear	0.21	2.01	Ref.	0.9474	0.6028
Anger	-8.63	-2.78	Ref.	0.0171	0.5208
Recognition	-3.65	-3.26	Ref.	0.0322	0.1111

[a,b,c] Superscript letters indicate significant differences among groups by Scheffe test for multiple comparison.
* Comparison of the percentage of correct answer of emotion recognition between groups adjusting for WAIS, age and sex by means of the general linear models.
SD: standard deviation; df: degrees of freedom; β: beta parameter estimated from the general linear model, considering the healthy controls (HC) as reference group; Ref: Reference group.
