# Supplementary material for: Differences in Facial Emotion Recognition between First Episode Psychosis, Borderline Personality Disorder and Healthy Controls
Source: PLoS One. 2016 Jul 28;11(7):e0160056. doi: 10.1371/journal.pone.0160056 (PMC4965014; doi:10.1371/journal.pone.0160056)
Supplement: S4 Table — (PDF) [file pone.0160056.s004.pdf]

Table 4. Comparison of the percentage of subjects' attribution when they failed in FEP, BPD and HC.

	FEP[a]	BPD[b]	HC[c]	Kruskal-Wallis test	
	mean (SD)	mean (SD)	mean (SD)	$\chi^2_{(df=2)}$	p-value
Neutral					
Happiness	10.3 (10.3)	12.3 (11.5)	11.3 (11.4)	0.77	0.6789
Negative valence	6.9 (8.4)	10.2 (10.2)[c]	5.6 (7.5)[b]	7.84	0.0198
Happiness					
Neutral	6 (7)	6.8 (7.3)	7.2 (7.7)	1.13	0.5688
Negative valence	3.2 (5.5)[c]	4 (6.8)[c]	1.3 (3.7)[a,b]	16.41	0.0003
Fear					
Neutral	33.9 (13.5)[c]	35 (14.6)	40.7 (15.2)[a]	11.08	0.0039
Happiness	3.3 (7.2)[c]	2.1 (3.2)	1.4 (3.3)[a]	10.35	0.0057
Anger	4.3 (6.6)	3.1 (4.9)	4.3 (6.3)	0.73	0.6941
Anger					
Neutral	18 (13.2)	13.1 (9.7)	17.1 (13.7)	3.13	0.2094
Happiness	2.6 (4.4)	3.2 (4.7)	3 (5.4)	0.89	0.6405
Fear	11.4 (10.7)[c]	10.4 (10.6)	8.2 (10.4)[a]	7.74	0.0209

[a,b,c] Superscript letters indicate significant differences among groups by Scheffe test for multiple comparison.
SD: standard deviation; df: degrees of freedom.
